# Implementing a dengue vaccination programme—who, where and how?

**DOI:** 10.1093/trstmh/try070

**Published:** 2018-07-17

**Authors:** Hannah E. Clapham, Bridget A. Wills

**Affiliations:** 1 Centre for Tropical Medicine and Global Health, Nuffield Department of Medicine, University of Oxford, Oxford, UK; 2 Oxford University Clinical Research Unit, Wellcome Trust Asia Program, Hospital for Tropical Diseases, Ho Chi Minh City, Vietnam

**Keywords:** dengue, dengue vaccination, policy, serological screening, vaccination strategy

## Abstract

The complex interaction between dengue viruses and the human immune system means that development of a safe, effective dengue vaccine was never going to be simple. The only currently licenced dengue vaccine (Dengvaxia^®^) does, indeed, have a complex immune profile depending on recipients’ immune status, meaning that use of this vaccine is not straightforward. This commentary reviews the recommendations for vaccine use to date, and discusses issues and opportunities related to the implementation of vaccination programmes in light of these recommendations. Future dengue vaccines may also have similar profiles, so it is vital that these issues are addressed now to ensure optimal use of vaccination in the fight against dengue globally.

Development of a safe effective dengue vaccine was always going to be difficult due to the complex interaction between dengue viruses (DENV) and the human immune system. Although all four DENV serotypes are capable of causing the full spectrum of clinical outcomes—ranging from asymptomatic infection through to severe and potentially fatal disease—a first/primary infection with any serotype rarely results in severe disease. Long-term protection is induced against this first serotype, but only short-term protection against the remaining three serotypes. During a second DENV infection with a different serotype a major risk factor for severe dengue is the presence of sub-neutralizing immunity to the previous virus, by a phenomenon known as antibody-dependent enhancement (ADE). Fortunately, severe disease is also uncommon with third and fourth DENV infections, suggesting that a broad and sustained immune response is achieved after the second exposure.

Concerns that ADE might occur following vaccination have been raised throughout the history of dengue vaccine development. However, the massive global dengue disease burden and the failure of existing vector-control measures to control transmission provide strong motivation to develop a vaccine suitable for widespread deployment. Dengvaxia^®^, Sanofi-Pasteur’s tetravalent live attenuated vaccine (given in three doses 6 months apart), was the first candidate to complete Phase 3 clinical trials,^1,2^ but with only moderate vaccine efficacy demonstrated and a suggestion of enhanced disease in previously dengue-naïve recipients and/or those under 9 years old. In 2016, based on these findings and related modelling work,^3^ the WHO recommended vaccination in those over 9 years old and in areas of ‘high burden’.^4,5^ High burden was defined as a seroprevalence greater than 70% and a guide was published describing how to undertake dengue serosurveys.^6^ Despite concerns,^7^ Dengvaxia^®^ progressed to licensure in a number of dengue-endemic countries, and large-scale vaccination programmes commenced in the Philippines and Brazil. However this all changed in November 2017, when evidence emerged from additional analyses^8^ and ongoing surveillance that enhanced disease was, indeed, occurring in dengue-naïve vaccine recipients in the trials. Sanofi-Pasteur issued a statement indicating that the vaccine should be used in those with a ‘high likelihood of a prior dengue infection’,^9^ and in their revised guidelines, issued in April 2018, WHO recommended a ‘pre-vaccination screening strategy’, i.e. testing of all potential vaccine recipients for anti-dengue antibody—to ensure that only dengue-seropositive persons are vaccinated.^10^

This new recommendation to vaccinate only those with documented prior dengue exposure needs to be considered in the context of dengue epidemiology and natural history. Since primary infections are typically asymptomatic or mild, determining past exposure does require formal serological confirmation. Currently a number of tests are available (indirect ELISA, haemagglutination inhibition or plaque reduction neutralization assays) with different performance characteristics and processing difficulty. When planning a country’s vaccination strategy therefore, factors such as serological test sensitivity, specificity, processing time and cost, must all be taken into account to ensure the most cost-effective use of limited resources.^11^ Of the assays available presently, only the indirect ELISA would be feasible at scale. However, there is increasing interest in the development of point-of-care tests that could potentially mitigate these costs.^12^ The need for an additional visit to communicate test results and vaccinate if indicated would also add considerably to the existing logistical challenges of administering a three-dose vaccination schedule to school-age children. Adding to the complexity, test sensitivity and specificity depend partly on local transmission of and/or vaccination against flaviviruses that cross-react with dengue, such as Japanese encephalitis (JE) and Zika. These interactions between naturally acquired flavivirus infections and vaccine-induced immune responses remain poorly understood, with more research needed to clearly elucidate the relationships and better define interactions with serodiagnostic test results.

For each country considering deployment of Dengvaxia^®^, complex decisions will be needed on the breadth and scope of any programme proposed. Individual serological testing will only be useful in certain settings, but how to decide where those are? Dengue transmission is notoriously variable over time and from place to place, so decisions must be taken on relevant spatial scales using information accrued over an appropriate time-period. In Vietnam for example, transmission of dengue is markedly heterogeneous across the country and, although JE vaccination has been widely promoted, uptake varies considerably in rural vs urban settings. Testing at too young an age for a given location will be costly with little benefit, since most individuals will turn out to be seronegative and thus ineligible for vaccination. In contrast, testing at too old an age for a given location will mean that, among seropositive individuals, many are likely to have had two or more prior dengue infections, and are thus already protected against severe disease. The previous WHO guidelines recommended performing serosurveys to ensure vaccination efforts were focused towards individuals with lower risk for ADE.^4,6^ In this new era, serosurveys could still be useful to:

- determine transmission intensity in different locations;
- inform decision-making regarding where and at what age individual testing-based vaccination programmes should be rolled out.

Importantly, data from serosurveys could also be used to optimize strategies for repeat testing. Re-testing of seronegative individuals (i.e. those not eligible for vaccination on first screening), must strike a balance between being so frequent as to incur the extra cost of multiple negative results, versus being so infrequent that the individual risks experiencing both primary and secondary exposures in the time interval between tests.

With all these difficulties in implementing such a vaccination programme, is it really worth it? Valuable lessons have been learnt from the Dengvaxia® experience, but two other vaccines are on the horizon. Should this vaccine just be chalked up to experience and should we move on? Views are mixed, with some saying it is time to move on, while others, including WHO, believe that there is still benefit in deploying Dengvaxia^®^ in semi-immune individuals, i.e. those at greatest risk of severe disease upon their next exposure. It should also be remembered that, since ADE remains a potential problem with the other dengue vaccines currently in development, it is likely these issues will remain relevant for some years to come. Therefore it would seem prudent to devote time and effort now on developing safe and effective vaccination strategies that take into account the potential for ADE, yet can be implemented practically in real-world settings. As has become clear in the Philippines, a dengue vaccination programme that is perceived as unsafe can seriously undermine public confidence in immunization practice, leading to reduced vaccine uptake in general.^13^ Careful consideration of how to implement a dengue vaccination programme, combined with transparency and good communication with the public and all major stakeholders, will be crucial to ensure that Dengvaxia^®^ and any other vaccines that proceed to licensure are deployed to maximal effect in the global fight against dengue.
